# Supplementary material for: Tissue-specific cell-free DNA degradation quantifies circulating tumor DNA burden
Source: Nat Commun. 2021 Apr 13;12:2229. doi: 10.1038/s41467-021-22463-y (PMC8044092; doi:10.1038/s41467-021-22463-y)
Supplement: Supplementary file 1 — Supplementary Information [file 41467_2021_22463_MOESM1_ESM.pdf]

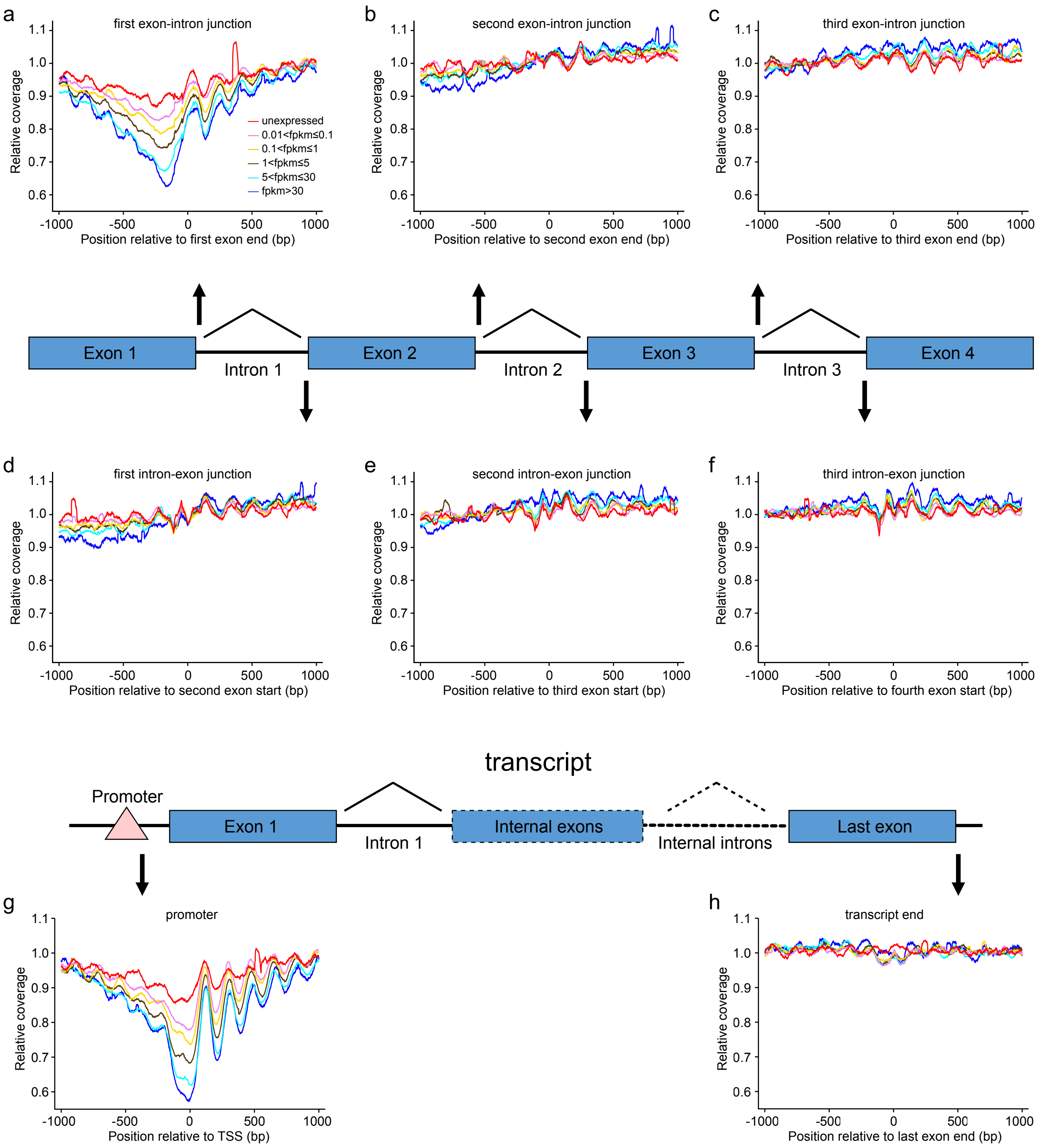

Supplementary Fig. 1 A systematic analysis of gene regions for association of gene expression and cfDNA relative coverage. Relative cfDNA coverage (normalized to +/-1kb flanking regions) for sets of genes grouped by expression level in whole blood cells across (**a**) first, (**b**) second, (**c**) third exon-intron junctions, (**d**) first, (**e**) second, (**f**) third intron-exon junctions, as well as (**g**) promoter, and (**h**) transcript end region.

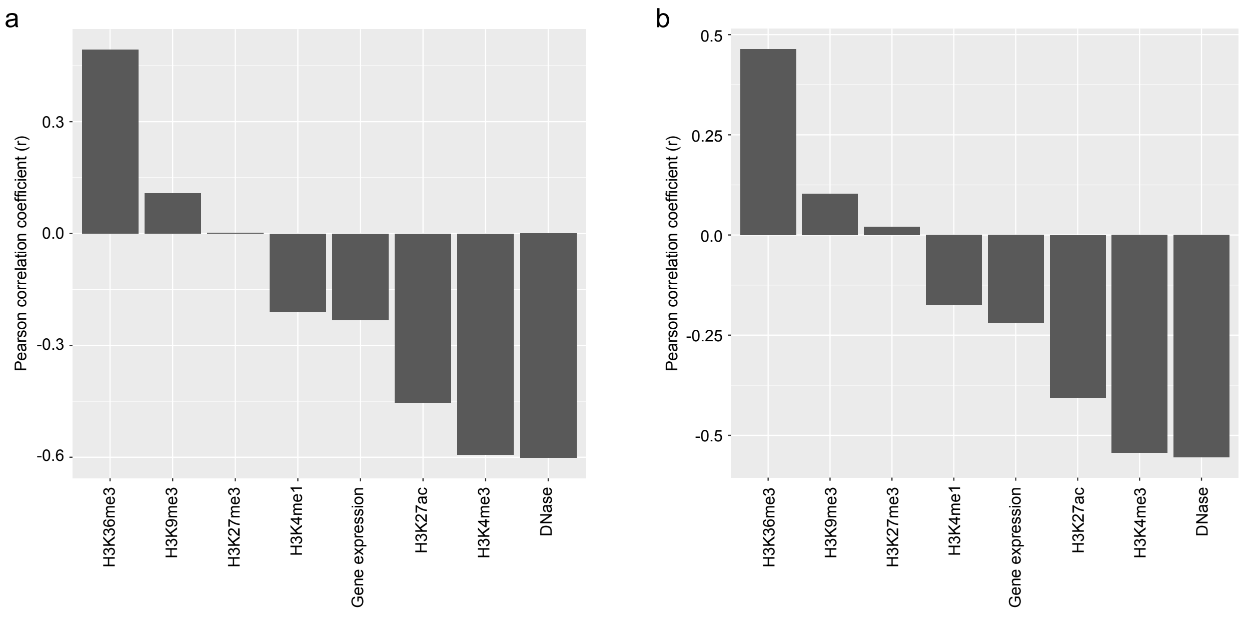

Supplementary Fig. 2 Correlation between relative coverage of NDRs and epigenetic features. For each candidate covariate/predictor, a linear regression is fitted with relative coverage as the response. The Pearson correlation coefficient (y axis, signed square root of R-squared from regression) is shown for each candidate variable. Whole blood gene expression (fpkm) is binned into 6 bins [unexpressed, $0.01 < fpkm \le 0.1$, $0.1 < fpkm \le 1$, $1 < fpkm \le 5$, $5 < fpkm \le 30$, $fpkm > 30$] and fitted as a categorical covariate with the unexpressed group as the reference group. Peak files of epigenetic features [DNase, H3K4me3, H3K36me3, H3K27ac, H3K4me1, H3K9me3 and H3K27me3] from primary T-cells (E034) were obtained from the Roadmap Epigenomics Project. Epigenetic features are fitted as binary covariates with no signal as the reference group. Barplots of the correlation (r, square root of R2 multiplied by the coefficient sign) between each feature and relative coverage for (**a**) promoter NDRs and (**b**) junction NDRs are shown.

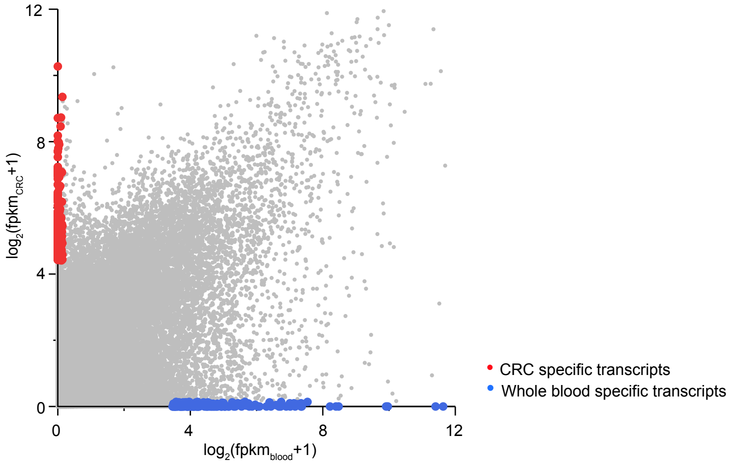

Supplementary Fig. 3 Transcripts differentially expressed between CRC tumors and whole blood. CRC ($fpkm_{CRC}$>20, $fpkm_{blood}$<0.1, red) and whole-blood ($fpkm_{CRC}$<0.1, $fpkm_{blood}$>10, blue) specific transcripts are identified from expression data from TCGA and GTEx.

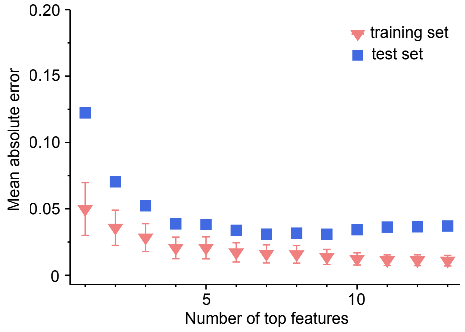

Supplementary Fig. 4 The evolution of predictive error with model complexity. Mean absolute error between observed and expected ctDNA fractions of CRC samples is estimated as a function of model complexity (number of top predictive features). The error bar size is the standard deviation of MAE values from 231 CRC training samples (pink).

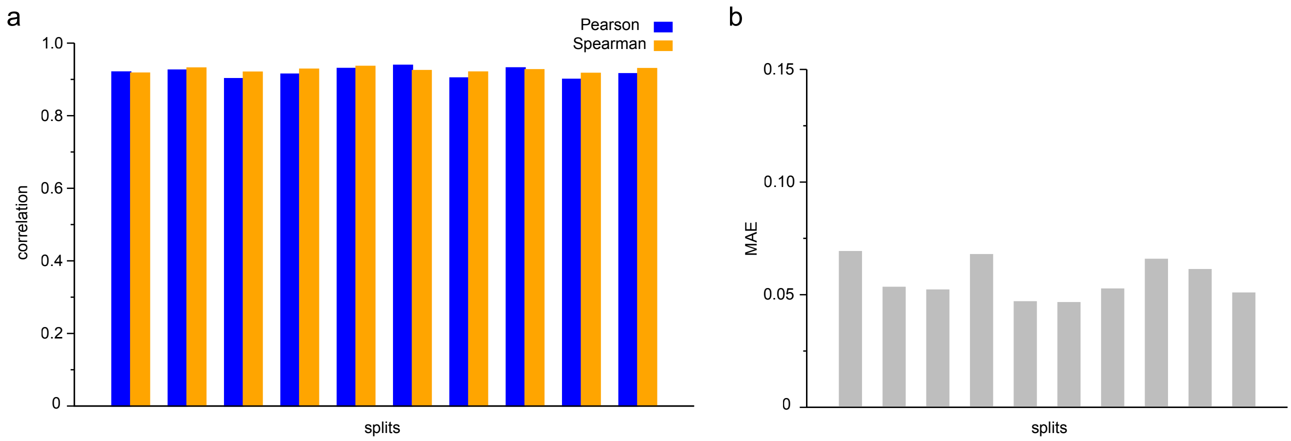

Supplementary Fig. 5 Model performance on 10 test sets generated using different (withheld) healthy samples from the training sets. Individual normal samples (n=29) in the healthy cohort were evenly split into 2 sets, used to dilute the plasma samples from CRC patients in training (CRC-1 to 8) and test (CRC-9 to 12) sets separately. (**a**) The correlation (Pearson and Spearman) between the expected and observed ctDNA fractions across the 10 test sets. (**b**) The mean absolute error (MAE) between the expected and observed ctDNA fractions for the 10 test sets.

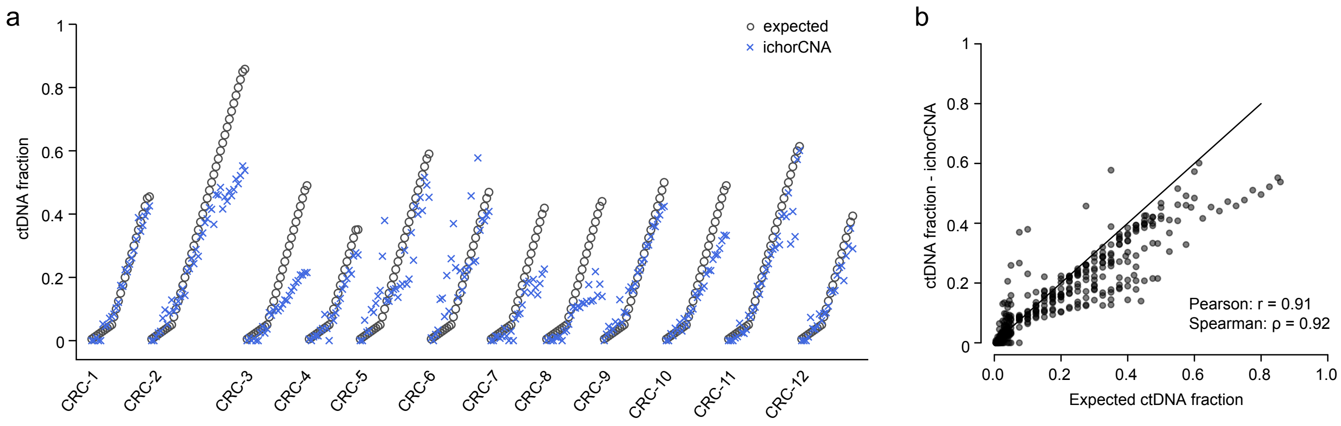

Supplementary Fig. 6 Comparison of expected and ichorCNA-predicted ctDNA fractions across the CRC cfDNA samples. (**a**) ctDNA fractions across the CRC cfDNA samples. (**b**) Comparison of expected and ichorCNA-predicted ctDNA fractions (n=344). The points are plotted in semi-transparent grey to indicate overlapping points.

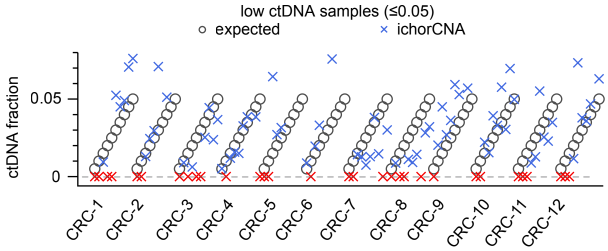

Supplementary Fig. 7 Performance of ichorCNA when applied to the samples with low ctDNA burden. 31 out of 120 low-ctDNA samples of CRC were predicted as non-cancerous by ichorCNA, highlighted in red. Grey dashed line indicates ctDNA fraction of 0.

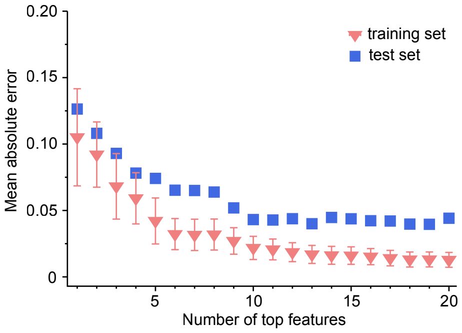

Supplementary Fig. 8 Predictive error as a function of model complexity for two distinct cancer types. The error bar size is the standard deviation of MAE values from 446 training samples (pink).

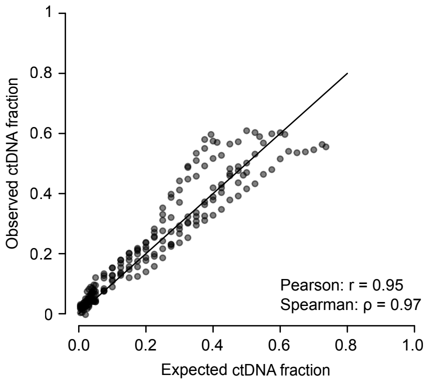

Supplementary Fig. 9 Comparison of expected and observed ctDNA fractions (n=206) in test set across two distinct cancer types. The points are plotted in semi-transparent grey to indicate overlapping points.

a

Gene	*Transcript*	*Chr*	*Site*	*Region*	*Expr.*	*FPKM$_{blood}$*	*FPKM$_{BRCA}$*	*Pr*
NUPR1	ENST00000395641	16	28550329	promoter	tumor	0.45	106.68	1
MLPH	ENST00000338530	2	238396080	junction	tumor	0.00	68.32	0.996
PTK7	ENST00000230419	6	43044305	junction	tumor	0.00	15.65	0.989
PROM2	ENST00000317620	2	95940577	junction	tumor	0.02	21.64	0.972
HID1	ENST00000425042	17	72968686	junction	tumor	0.23	38.07	0.968
TPD52	ENST00000379097	8	80992550	junction	tumor	0.50	70.34	0.961
SORD	ENST00000267814	15	45315547	junction	tumor	0.93	11.37	0.960
HOXC10	ENST00000303460	12	54378970	promoter	tumor	0.00	12.67	0.952

b

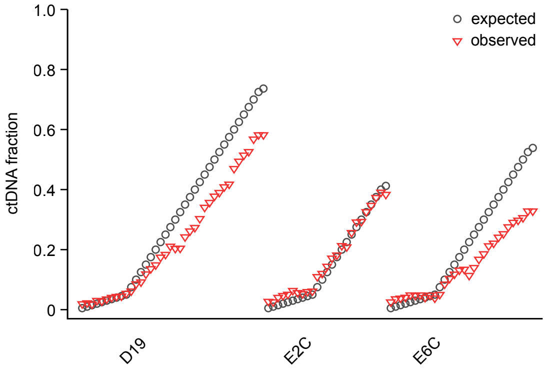

c

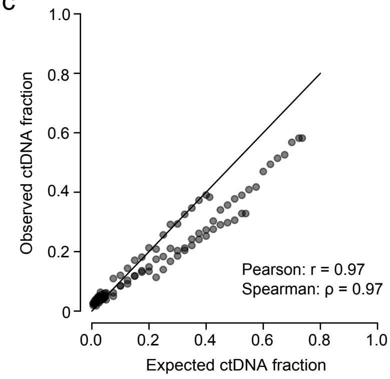

Supplementary Fig. 10 A BRCA model using BRCA tumor-specific NDRs. (**a**) The list of top BRCA tumor-specific NDRs that were used for ctDNA content prediction. (**b**,**c**) Comparison of expected and observed ctDNA fractions across the BRCA cfDNA samples (n=93) in the test set. The points are plotted in semi-transparent grey to indicate overlapping points.

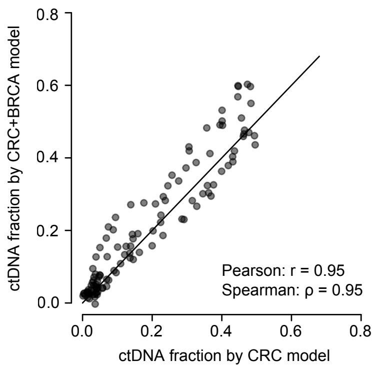

Supplementary Fig. 11 Comparison of the ctDNA fractions determined by the CRC model and the "CRC+BRCA" model for the CRC samples (n=113) in the test set. The points are plotted in semi-transparent grey to indicate overlapping points.

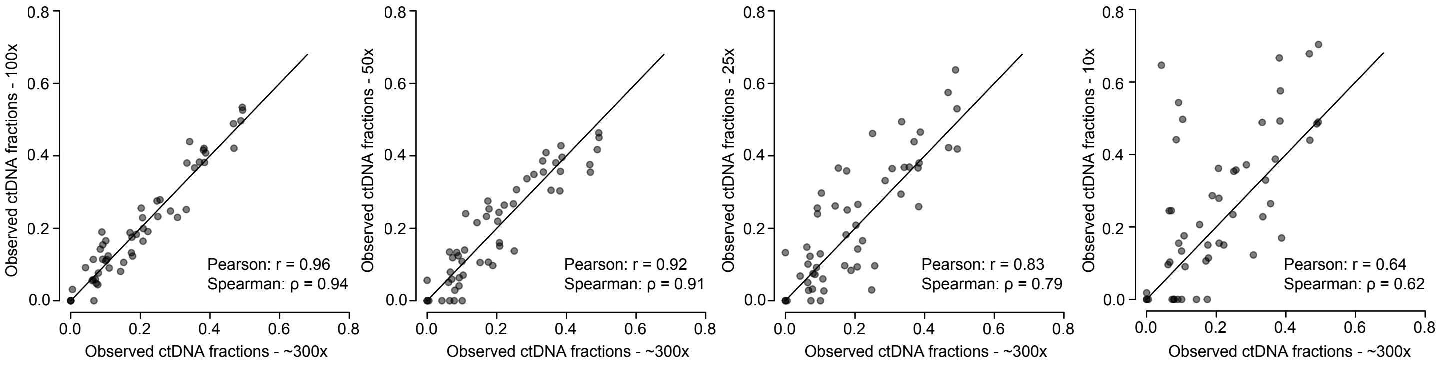

Supplementary Fig. 12 Comparison of the observed ctDNA fractions in the 53 original cfDNA samples with capture-based NDR sequencing (mean coverage ~300x) and their downsampled counterparts (100x, 50x, 25x, and 10x, respectively). The points are plotted in semi-transparent grey to indicate overlapping points.

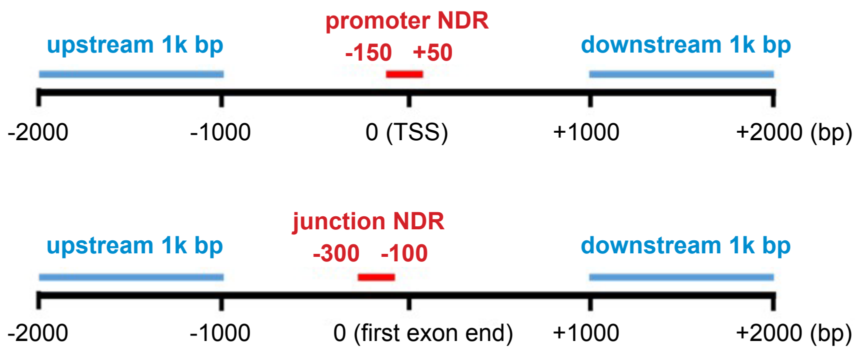

Supplementary Fig. 13 Genomic regions over promoters (top) and first exon-intron junction (bottom) used to calculate relative coverage. The mean coverage of the up and downstream 2kbp flanks (blue) is used as a “normalization factor” for the region of interest (red).

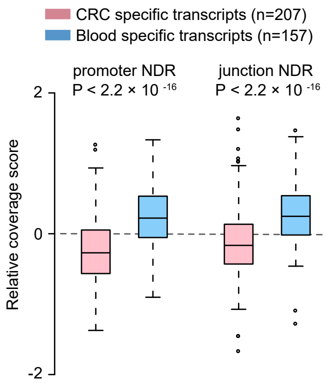

Supplementary Fig. 14 Relative coverage scores based on the variance of 20 healthy control subsets for the transcripts differentially expressed between CRC tumors and whole blood. Two-sided Wilcoxon rank-sum tests were performed to compare CRC and blood specific transcripts. Boxplots represent the median as centreline, the interquartile range (IQR) as bounds of box, and the lower quartile –1.5 IQR and the upper quartile +1.5 IQR as whiskers.

	patient ID	sample name	sample ID	ctDNA fraction	sample size
training set CRC	1014	1014_180816	CRC-1	0.005, 0.010, 0.015, 0.020, 0.025, 0.030, 0.035, 0.040, 0.045, 0.050, 0.075, 0.100, 0.125, 0.150, 0.175, 0.200, 0.225, 0.250, 0.275, 0.300, 0.325, 0.350, 0.375, 0.400, 0.425, 0.450, 0.455	27
	1531	1531_160616	CRC-2	0.005, 0.010, 0.015, 0.020, 0.025, 0.030, 0.035, 0.040, 0.045, 0.050, 0.075, 0.100, 0.125, 0.150, 0.175, 0.200, 0.225, 0.250, 0.275, 0.300, 0.325, 0.350, 0.375, 0.400, 0.425, 0.450, 0.475, 0.500, 0.525, 0.550, 0.575, 0.600, 0.625, 0.650, 0.675, 0.700, 0.725, 0.750, 0.775, 0.800, 0.825, 0.850, 0.858	43
	1531	1531_180119	CRC-3	0.005, 0.010, 0.015, 0.020, 0.025, 0.030, 0.035, 0.040, 0.045, 0.050, 0.075, 0.100, 0.125, 0.150, 0.175, 0.200, 0.225, 0.250, 0.275, 0.300, 0.325, 0.350, 0.375, 0.400, 0.425, 0.450, 0.475, 0.490	28
	519	519_210114	CRC-4	0.005, 0.010, 0.015, 0.020, 0.025, 0.030, 0.035, 0.040, 0.045, 0.050, 0.075, 0.100, 0.125, 0.150, 0.175, 0.200, 0.225, 0.250, 0.275, 0.300, 0.325, 0.350, 0.351	23
	809	809_030915	CRC-5	0.005, 0.010, 0.015, 0.020, 0.025, 0.030, 0.035, 0.040, 0.045, 0.050, 0.075, 0.100, 0.125, 0.150, 0.175, 0.200, 0.225, 0.250, 0.275, 0.300, 0.325, 0.350, 0.375, 0.400, 0.425, 0.450, 0.475, 0.500, 0.525, 0.550, 0.575, 0.590	32
	809	809_110914	CRC-6	0.005, 0.010, 0.015, 0.020, 0.025, 0.030, 0.035, 0.040, 0.045, 0.050, 0.075, 0.100, 0.125, 0.150, 0.175, 0.200, 0.225, 0.250, 0.275, 0.300, 0.325, 0.350, 0.375, 0.400, 0.425, 0.450, 0.469	27
	986	986_100215	CRC-7	0.005, 0.010, 0.015, 0.020, 0.025, 0.030, 0.035, 0.040, 0.045, 0.050, 0.075, 0.100, 0.125, 0.150, 0.175, 0.200, 0.225, 0.250, 0.275, 0.300, 0.325, 0.350, 0.375, 0.400, 0.419	25
	986	986_261016	CRC-8	0.005, 0.010, 0.015, 0.020, 0.025, 0.030, 0.035, 0.040, 0.045, 0.050, 0.075, 0.100, 0.125, 0.150, 0.175, 0.200, 0.225, 0.250, 0.275, 0.300, 0.325, 0.350, 0.375, 0.400, 0.425, 0.440	26
training set BRCA	BRCA-D14	D14	BRCA-1	0.005, 0.010, 0.015, 0.020, 0.025, 0.030, 0.035, 0.040, 0.045, 0.050, 0.075, 0.100, 0.125, 0.150, 0.175, 0.200, 0.225, 0.250, 0.275, 0.300, 0.325, 0.350, 0.375, 0.400, 0.425, 0.450, 0.475, 0.500, 0.525, 0.550, 0.575, 0.600, 0.625, 0.650, 0.675, 0.6967	36
	BRCA-D23	D23	BRCA-2	0.005, 0.010, 0.015, 0.020, 0.025, 0.030, 0.035, 0.040, 0.045, 0.050, 0.075, 0.100, 0.125, 0.150, 0.175, 0.200, 0.225, 0.250, 0.275, 0.300, 0.325, 0.350, 0.375, 0.400, 0.425, 0.450, 0.475, 0.500, 0.525, 0.550, 0.575, 0.600, 0.625, 0.650, 0.675, 0.700, 0.725, 0.750, 0.7567	39
	BRCA-D7	D7	BRCA-3	0.005, 0.010, 0.015, 0.020, 0.025, 0.030, 0.035, 0.040, 0.045, 0.050, 0.075, 0.100, 0.125, 0.1268	14
	BRCA-D9	D9	BRCA-4	0.005, 0.010, 0.015, 0.020, 0.025, 0.030, 0.035, 0.040, 0.045, 0.050, 0.075, 0.100, 0.125, 0.150, 0.175, 0.200, 0.225, 0.250, 0.275, 0.300, 0.325, 0.350, 0.375, 0.400, 0.425, 0.450, 0.475, 0.500, 0.525, 0.550, 0.575, 0.600, 0.625, 0.650, 0.6577	35
	BRCA-E10c	E10c	BRCA-5	0.005, 0.010, 0.015, 0.020, 0.025, 0.030, 0.035, 0.040, 0.045, 0.050, 0.075, 0.100, 0.125, 0.150, 0.175, 0.200, 0.225, 0.250, 0.275, 0.300, 0.325, 0.350, 0.375, 0.400, 0.425, 0.450, 0.475, 0.500, 0.525, 0.550, 0.575, 0.5931	32
	BRCA-E7c	E7c	BRCA-6	0.005, 0.010, 0.015, 0.020, 0.025, 0.030, 0.035, 0.040, 0.045, 0.050, 0.075, 0.100, 0.125, 0.150, 0.175, 0.200, 0.225, 0.250, 0.275, 0.300, 0.325, 0.350, 0.375, 0.400, 0.425, 0.450, 0.475, 0.500, 0.5078	29
	BRCA-E8c	E8c	BRCA-7	0.005, 0.010, 0.015, 0.020, 0.025, 0.030, 0.035, 0.040, 0.045, 0.050, 0.075, 0.100, 0.125, 0.150, 0.175, 0.200, 0.225, 0.250, 0.275, 0.300, 0.325, 0.350, 0.375, 0.400, 0.425, 0.450, 0.475, 0.500, 0.525, 0.5356	30

test set CRC	1279	1279_221015	CRC-9	0.005, 0.010, 0.015, 0.020, 0.025, 0.030, 0.035, 0.040, 0.045, 0.050, 0.075, 0.100, 0.125, 0.150, 0.175, 0.200, 0.225, 0.250, 0.275, 0.300, 0.325, 0.350, 0.375, 0.400, 0.425, 0.450, 0.475, 0.500	28
	1279	1279_241116	CRC-10	0.005, 0.010, 0.015, 0.020, 0.025, 0.030, 0.035, 0.040, 0.045, 0.050, 0.075, 0.100, 0.125, 0.150, 0.175, 0.200, 0.225, 0.250, 0.275, 0.300, 0.325, 0.350, 0.375, 0.400, 0.425, 0.450, 0.475, 0.490	28
	512	512_051015	CRC-11	0.005, 0.010, 0.015, 0.020, 0.025, 0.030, 0.035, 0.040, 0.045, 0.050, 0.075, 0.100, 0.125, 0.150, 0.175, 0.200, 0.225, 0.250, 0.275, 0.300, 0.325, 0.350, 0.375, 0.400, 0.425, 0.450, 0.475, 0.500, 0.525, 0.550, 0.575, 0.600, 0.614	33
	512	512_130114	CRC-12	0.005, 0.010, 0.015, 0.020, 0.025, 0.030, 0.035, 0.040, 0.045, 0.050, 0.075, 0.100, 0.125, 0.150, 0.175, 0.200, 0.225, 0.250, 0.275, 0.300, 0.325, 0.350, 0.375, 0.394	24
test set BRCA	D19	D19	BRCA-8	0.005, 0.010, 0.015, 0.020, 0.025, 0.030, 0.035, 0.040, 0.045, 0.050, 0.075, 0.100, 0.125, 0.150, 0.175, 0.200, 0.225, 0.250, 0.275, 0.300, 0.325, 0.350, 0.375, 0.400, 0.425, 0.450, 0.475, 0.500, 0.525, 0.550, 0.575, 0.600, 0.625, 0.650, 0.675, 0.700, 0.725, 0.7363	38
	E2c	E2c	BRCA-9	0.005, 0.010, 0.015, 0.020, 0.025, 0.030, 0.035, 0.040, 0.045, 0.050, 0.075, 0.100, 0.125, 0.150, 0.175, 0.200, 0.225, 0.250, 0.275, 0.300, 0.325, 0.350, 0.375, 0.400, 0.4122	25
	E6c	E6c	BRCA-10	0.005, 0.010, 0.015, 0.020, 0.025, 0.030, 0.035, 0.040, 0.045, 0.050, 0.075, 0.100, 0.125, 0.150, 0.175, 0.200, 0.225, 0.250, 0.275, 0.300, 0.325, 0.350, 0.375, 0.400, 0.425, 0.450, 0.475, 0.500, 0.525, 0.5386	30

Supplementary Table 1 The *in silico* samples of various ctDNA content.

CRC model					
feature	gene	transcript	region	group	coefficient
1	*SHKBP1*	ENST00000599716	junction	blood	0.607
2	*ACSL1*	ENST00000454703	junction	blood	0.431
3	*BCAR1*	ENST00000162330	junction	tumor	-0.321
4	*RAB25*	ENST00000361084	promoter	tumor	-0.213
5	*PRTN3*	ENST00000234347	promoter	blood	0.062
6	*LSR*	ENST00000605618	promoter	tumor	-0.174
CRC+BRCA model					
feature	gene	transcript	region	group	coefficient
1	*SLC11A1*	ENST00000465984	promoter	blood	0.150
2	*NLRP12*	ENST00000324134	promoter	blood	0.181
3	*PRTN3*	ENST00000234347	promoter	blood	0.124
4	*HMBS*	ENST00000392841	promoter	blood	0.251
5	*LILRB3*	ENST00000460208	promoter	blood	0.140
6	*ACSL1*	ENST00000513001	junction	blood	0.106
7	*GP9*	ENST00000307395	junction	blood	0.251
8	*MX2*	ENST00000398632	promoter	blood	0.106
9	*RASGRP4*	ENST00000615340	promoter	blood	0.222
10	*ATG16L2*	ENST00000542481	promoter	blood	0.166

Supplementary Table 2 Coefficients for the selected NDRs in the trained models. The intercept values are 0.4368 and -1.3719 for CRC and CRC+BRCA models respectively.

source	tissue	sample size	download URLs
GTEx	whole blood	337	https://toil.xenahubs.net/download/gtex_RSEM_isoform_fpkm.gz
TCGA	tumor of colorectal cancer (CRC)	372	https://toil.xenahubs.net/download/tcga_RSEM_isoform_fpkm.gz

Supplementary Table 3 Transcript expression data.
