# Supplementary material for: Tissue-specific cell-free DNA degradation quantifies circulating tumor DNA burden
Source: Nat Commun. 2021 Apr 13;12:2229. doi: 10.1038/s41467-021-22463-y (PMC8044092; doi:10.1038/s41467-021-22463-y)
Supplement: Supplementary file 3 — Description of Additional Supplementary Files [file 41467_2021_22463_MOESM3_ESM.docx]

**Description of Additional Supplementary Files**

File Name: Supplementary Data 1

Description: ctDNA burden estimation of plasma samples from cancer patients

File Name: Supplementary Data 2

Description: Information on all candidate features of nucleosome-depleted regions for colorectal cancer

File Name: Supplementary Data 3

Description: Observed ctDNA fractions in the LOD analysis for the CRC model

File Name: Supplementary Data 4

Description: CRC plasma samples for lp-WGS and targeted sequencing

File Name: Supplementary Data 5

Description: A panel of 100 genes frequently mutated in colorectal cancer

File Name: Supplementary Data 6

Description: Variant allele frequency estimation of plasma samples from CRC patients

File Name: Supplementary Data 7

Description: Mutations missed by the callers for the CRC patients with serial plasma samples

File Name: Supplementary Data 8

Description: Information on all candidate pan-cancer features of nucleosome-depleted regions

File Name: Supplementary Data 9

Description: Top 10 predictive features in the CRC+BRCA model

File Name: Supplementary Data 10

Description: Observed ctDNA fractions in the LOD analysis for the CRC+BRCA model

File Name: Supplementary Data 11

Description: Information of patients in the study

File Name: Supplementary Data 12

Description: Specific locations corresponding to the genes frequently mutated in colorectal cancer (100_gene_panel_hg38)

File Name: Supplementary Data 13

Description: A panel of 77 genes for screening breast cancer samples

File Name: Supplementary Data 14

Description: Specific locations corresponding to the genes frequently mutated in breast cancer (77_gene_panel_hg38)

File Name: Supplementary Data 15

Description: Probes designed for targeted NDR sequencing

File Name: Supplementary Data 16

Description: The samples with NDR-positive ctDNA detection but SNV and ichorCNA-negative

File Name: Supplementary Software 1

Description: The code for generating coverage features and developing quantitative models
