# Supplementary material for: Tissue-specific cell-free DNA degradation quantifies circulating tumor DNA burden
Source: Nat Commun. 2021 Apr 13;12:2229. doi: 10.1038/s41467-021-22463-y (PMC8044092; doi:10.1038/s41467-021-22463-y)
Supplement: Supplementary file 21 — Reporting Summary [file 41467_2021_22463_MOESM21_ESM.pdf]

nature research

Corresponding author(s): Anders Jacobsen Skanderup
Last updated by author(s): Jan 20, 2021

Reporting Summary

Nature Research wishes to improve the reproducibility of the work that we publish. This form provides structure for consistency and transparency in reporting. For further information on Nature Research policies, see our Editorial Policies and the Editorial Policy Checklist.

Statistics

For all statistical analyses, confirm that the following items are present in the figure legend, table legend, main text, or Methods section.

n/a	Confirmed	
☐	☒	The exact sample size (*n*) for each experimental group/condition, given as a discrete number and unit of measurement
☐	☒	A statement on whether measurements were taken from distinct samples or whether the same sample was measured repeatedly
☐	☒	The statistical test(s) used AND whether they are one- or two-sided<br>*Only common tests should be described solely by name; describe more complex techniques in the Methods section.*
☐	☒	A description of all covariates tested
☐	☒	A description of any assumptions or corrections, such as tests of normality and adjustment for multiple comparisons
☐	☒	A full description of the statistical parameters including central tendency (e.g. means) or other basic estimates (e.g. regression coefficient) AND variation (e.g. standard deviation) or associated estimates of uncertainty (e.g. confidence intervals)
☐	☒	For null hypothesis testing, the test statistic (e.g. *F*, *t*, *r*) with confidence intervals, effect sizes, degrees of freedom and *P* value noted<br>*Give P values as exact values whenever suitable.*
☒	☐	For Bayesian analysis, information on the choice of priors and Markov chain Monte Carlo settings
☒	☐	For hierarchical and complex designs, identification of the appropriate level for tests and full reporting of outcomes
☐	☒	Estimates of effect sizes (e.g. Cohen's *d*, Pearson's *r*), indicating how they were calculated

Our web collection on statistics for biologists contains articles on many of the points above.

Software and code

Policy information about availability of computer code

Data collection	Sequencing data in fastq format was collected by Illumina instruments
Data analysis	The code for generating coverage features and developing quantitative models is included as supplementary software. The NDR models, code, and data accessions are available at https://github.com/skandlab/NDRquant.<br><br>We used R glmnet for the machine learning modelling (v3)<br>bwa mem was used for aligning reads (v0.7.17)<br>samtools was used for sorting and merging bam files, calculating coverage, as well as downsampling sequence reads (v1.7)<br>bamUtil was used for trimming reads (v1.0.14)<br>biobambam was used for marking duplicates (https://github.com/gt1/biobambam)<br>MuTect was used for mutation calling (version 1.1.7)<br>VarScan was used for mutation calling (version 2.4.2)<br>GATK was used for recalibration and realignment (version 3.7)<br>Variant Effect Predictor was used for annotating all calls (version 87)<br>ichorCNA was used for estimate ctDNA fraction (https://github.com/broadinstitute/ichorCNA)<br>deep WGS data was analyzed using the bcbio-nextgen workflow that internally comprises multiple-step analysis (v1.0.7)

For manuscripts utilizing custom algorithms or software that are central to the research but not yet described in published literature, software must be made available to editors and reviewers. We strongly encourage code deposition in a community repository (e.g. GitHub). See the Nature Research guidelines for submitting code & software for further information.

Data

Policy information about availability of data

All manuscripts must include a data availability statement. This statement should provide the following information, where applicable:

- Accession codes, unique identifiers, or web links for publicly available datasets
- A list of figures that have associated raw data
- A description of any restrictions on data availability

cfDNA sequencing data have been deposited at the European Genome-phenome Archive (EGA) under the accession code EGAS00001004657 (https://ega-archive.org/studies/EGAS00001004657). The data is made available for academic research. Data will be released subject to a data transfer agreement. Tissue-specific RNA-seq transcript expression data (https://toil.xenahubs.net/download/gtex_RSEM_isoform_fpkm.gz) based on GTEx dataset and tumor RNA-seq transcript expression data (https://toil.xenahubs.net/download/tcga_RSEM_isoform_fpkm.gz) based on TCGA dataset were obtained from UCSC Toil RNAseq Recompute Compendium.

Field-specific reporting

Please select the one below that is the best fit for your research. If you are not sure, read the appropriate sections before making your selection.

☒ Life sciences ☐ Behavioural & social sciences ☐ Ecological, evolutionary & environmental sciences

For a reference copy of the document with all sections, see nature.com/documents/nr-reporting-summary-flat.pdf

Life sciences study design

All studies must disclose on these points even when the disclosure is negative.

Sample size	The cohort contains 29 plasma cfDNA samples from healthy individuals, 12 plasma cfDNA samples from colorectal cancer patients, and 10 plasma cfDNA samples from breast cancer patients. The 12 colorectal and 10 breast cancer plasma samples were identified with high SNV VAFs (indicating high ctDNA burden), thus we performed deep WGS to discover the NDRs predictive of ctDNA burden. To the best of our knowledge, we generated the largest existing cfDNA deep-WGS dataset, comprising ~90x cfDNA cancer genomes of 12 colorectal and 10 breast cancer cfDNA samples, and we think the current dataset is sufficient to develop a robust model.
Data exclusions	There were no data exclusions.
Replication	Due to the limited volumes of plasma available per patient, it was not possible to perform the sequencing assay more than once for each sample.<br><br>However, in our computational analysis, we repeated the modeling for multiple times in order to identify robust features and evaluate the robustness of the model:<br>To select robust features, we first extracted half of the training data randomly and used Lasso with ten-fold cross-validation to identify features predictive of ctDNA fractions. This procedure was repeated 1000 times to identify the robust predictive features (selection frequency ≥ 0.99). Besides, we demonstrated the robustness of the model when we trained and tested on in silico samples generated using independent healthy samples. To measure reproducibility, we repeated this procedure 10 times with sample replacement and all replicas confirmed the model accuracy.
Randomization	This is not a randomized study, thus no randomization has been performed.
Blinding	Blinding was not applied in our study, because plasma samples from all cancer patients were included for model development and validation regardless of treatment protocol.

Reporting for specific materials, systems and methods

We require information from authors about some types of materials, experimental systems and methods used in many studies. Here, indicate whether each material, system or method listed is relevant to your study. If you are not sure if a list item applies to your research, read the appropriate section before selecting a response.

Materials & experimental systems

n/a	Involved in the study	
x		Antibodies
x		Eukaryotic cell lines
x		Palaeontology and archaeology
x		Animals and other organisms
	x	Human research participants
x		Clinical data
x		Dual use research of concern

Methods

n/a	Involved in the study	
x		ChIP-seq
x		Flow cytometry
x		MRI-based neuroimaging

Human research participants

Policy information about studies involving human research participants

Population characteristics	A metadata comprising population characteristics is available in Supplementary Data 1.
Recruitment	the patients from the hospital
Ethics oversight	Singhealth Centralised Institutional Review Board

Note that full information on the approval of the study protocol must also be provided in the manuscript.
